# Posterior reversible leukoencephalopathy syndrome associated with acute postinfectious glomerulonephritis: systematic review

**DOI:** 10.1007/s00467-021-05244-z

**Published:** 2021-09-21

**Authors:** Corinne Orlando, Gregorio P. Milani, Giacomo D. Simonetti, Barbara Goeggel Simonetti, Sebastiano A. G. Lava, Rolf Wyttenbach, Mario G. Bianchetti, Marirosa Cristallo Lacalamita

**Affiliations:** 1 Family Medicine Institute, Faculty of Biomedical Sciences, Università Della Svizzera Italiana, Lugano, Switzerland; 2 Pediatric Unit, Fondazione IRCCS Ca’ Granda Ospedale Maggiore Policlinico, Milan, Italy; 3 Department of Clinical Sciences and Community Health, Università Degli Studi Di Milano, Milan, Italy; 4 Pediatric Institute of Southern Switzerland EOC, Ospedale San Giovanni, Bellinzona, Switzerland; 5 Faculty of Biomedical Sciences, Università Della Svizzera Italiana, Lugano, Switzerland; 6 Department of Neurology, University Hospital Bern, University of Bern, Bern, Switzerland; 7 Pediatric Cardiology Unit, Department of Pediatrics, Centre Hospitalier Universitaire Vaudois and University of Lausanne, Lausanne, Switzerland; 8 Imaging Institute of Southern Switzerland EOC, Bellinzona, Switzerland; 9 Department of Diagnostic, Interventional and Pediatric Radiology (DIPR), Inselspital, Bern University Hospital, University of Bern, Bern, Switzerland

**Keywords:** Acute brain capillary leak syndrome, Acute postinfectious glomerulonephritis, Acute poststreptococcal glomerulonephritis, Hypertensive encephalopathy, Posterior reversible leukoencephalopathy syndrome

## Abstract

**Background:**

Kidney diseases are a recognized cause of posterior reversible leukoencephalopathy syndrome, usually abbreviated as PRES. The purpose of this review was to systematically address the association between acute postinfectious glomerulonephritis and PRES.

**Methods:**

We performed a systematic review of the literature on acute postinfectious glomerulonephritis associated with PRES. The principles recommended by the Economic and Social Research Council guidance on the conduct of narrative synthesis and on the Preferred Reporting Items for Systematic Reviews and Meta-analyses were used. Databases searched included Excerpta Medica, US National Library of Medicine, and Web of Science.

**Results:**

For the final analysis, we evaluated 47 reports describing 52 cases (32 males and 20 females). Fifty patients were ≤ 18 years of age. Blood pressure was classified as follows: normal-elevated (*n* = 3), stage 1 hypertension (*n* = 3), stage 2 hypertension (*n* = 5), and severe hypertension (*n* = 41). Acute kidney injury was classified as stage 1 in 32, stage 2 in 16, and stage 3 in four cases. Neuroimaging studies disclosed a classic posterior PRES pattern in 28 cases, a diffuse PRES pattern in 23 cases, and a brainstem-cerebellum PRES pattern in the remaining case. Antihypertensive drugs were prescribed in all cases and antiepileptic drugs in cases presenting with seizures. A resolution of clinical findings and neuroimaging lesions was documented in all cases with information about follow-up.

**Conclusions:**

The main factor associated with PRES in acute postinfectious glomerulonephritis is severe hypertension. Prompt clinical suspicion, rapid evaluation, and management of hypertension are crucial.

**Graphical abstract:**

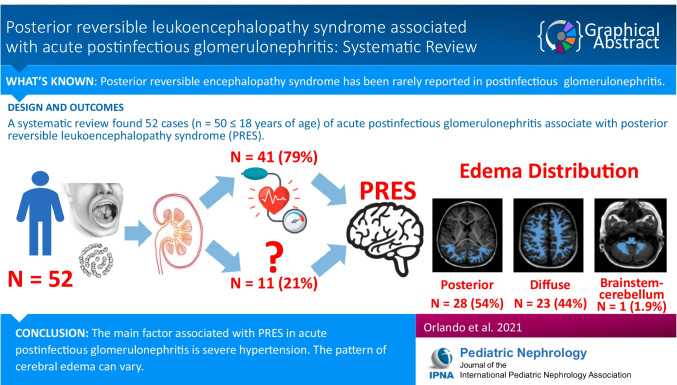

A higher resolution version of the Graphical abstract is available as Supplementary information

**Supplementary Information:**

The online version contains supplementary material available at 10.1007/s00467-021-05244-z.

## Introduction

Posterior reversible leukoencephalopathy syndrome, usually abbreviated as PRES, is a rather uncommon condition, whose diagnosis relies both on clinical and imaging features [1, 2]. The clinical presentation includes headache or nausea; altered mental status; seizures; visual perception abnormalities; and, less frequently, further focal neurologic signs. Neuroimaging studies demonstrate a characteristically reversible vasogenic edema that generally involves the subcortical white matter in the posterior cerebral regions [2]. A variety of conditions have been implicated as causes of PRES, including among others severe arterial hypertension and both acute and chronic kidney diseases [2]. Some drugs have also been deemed to cause PRES [3].

Stimulated by our past experience [4, 5], we report here the first systematic literature review on PRES in postinfectious (poststreptococcal) glomerulonephritis, the prototypical and most widely known acute glomerulonephritis syndrome [6, 7]. The objectives were to document features, associated factors, neuroimaging data, and clinical course.

## Methods

### Literature search strategy

For this analysis, we followed the Preferred Reporting Items for Systematic Reviews and Meta-Analyses guidelines and searched up to November 2020 in the US National Library of Medicine, Excerpta Medica, and Web of Sciences databases [8]. We carried out a structured literature search without date limitation using the Medical Subject Headings terms “poststreptococcal glomerulonephritis” OR “postinfectious glomerulonephritis” AND “encephalopathy” OR “posterior reversible encephalopathy syndrome” OR “posterior reversible leukoencephalopathy syndrome” OR “PRES.” Articles in languages other than Spanish, Portuguese, Italian, German, French, English, or Dutch were not included. The literature search was carried out by two investigators (CO, MGB), who independently screened the title and abstract of all reports in a non-blinded fashion to include pertinent reports. Discrepancies in study identification were resolved by consensus. Subsequently, full-text publications were reviewed to decide whether the presented case fit the eligibility criteria of the review. Personal files and the bibliography of each identified report were also screened for further references. Institutional Review Board approval was not required for this literature review.

### Selection criteria—definitions—data extraction

Of interest were subjects without any pre-existing kidney or cerebral disease presenting with acute postinfectious glomerulonephritis and PRES, whose history had been published in full-length articles or letters. Prospectively defined criteria were used to confirm or infirm the diagnosis of acute poststreptococcal glomerulonephritis, arterial hypertension, and PRES made in the original articles.

The diagnosis of postinfectious glomerulonephritis was retained in patients with acute onset of hematuria and proteinuria associated with (a) C_3_ hypocomplementemia returning to normal within 16 weeks, (b) increasing anti-streptoscorecoccal antibodies, or (c) distinctive kidney biopsy findings [6, 7]. The KDIGO criteria [9] were used to classify acute kidney damage as stage 1 (increase in creatinine to 1.5–1.9 times baseline or increase by ≥ 27 μmol/l above the upper limit of normal for age), stage 2 (increase in creatinine to 2.0–2.9 times baseline), or stage 3 (increase in creatinine to ≥ 3.0 times baseline, increase in creatinine by ≥ 354 μmol/l, or initiation of dialysis).

The highest recorded blood pressure value at presentation was used to categorize hypertension as stage 1 (subjects 1 to < 13 years of age with blood pressure readings ≥ 95th percentile to 95th percentile + 12 mm Hg; subjects ≥ 13 years with readings between 130/80 and 139/89 mm Hg); stage 2 (subjects 1 to < 13 years of age with readings 95th percentile + 12 mm Hg to 95th percentile + 30 mm Hg; subjects aged ≥ 13 years with reading ≥ 140/90 to 179/119 mm Hg); or severe (subjects 1 to < 13 years of age with readings ≥ 95th percentile +  > 30 mm Hg; subjects aged ≥ 13 years with readings > 180/120 mm Hg). Blood pressure was categorized as normal in the remaining cases [10, 11].

The diagnosis of PRES was made on the basis of both an acute onset of at least one of five neurologic features ((1) headache or nausea; (2) altered mental status; (3) seizures; (4) visual perception abnormalities; (5) further focal neurologic signs) and distinctive neuroimaging findings [12, 13].

**Fig. 1:**
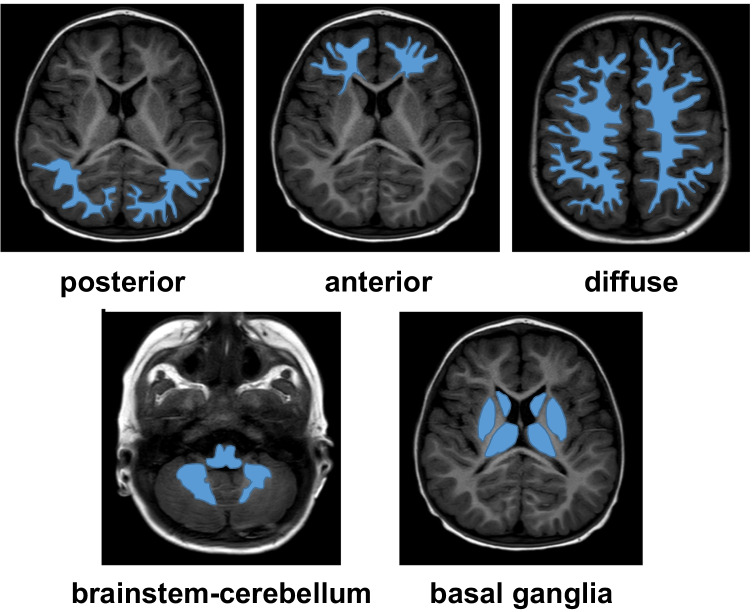
Posterior reversible leukoencephalopathy syndrome. Sketch depicting the five lesion location patterns of white matter edema (blue): posterior, anterior, diffuse, brainstem-cerebellum, and basal ganglia

Neuroimaging data were classified based on lesion location patterns and severity. Lesion location patterns were categorized based on cerebral edema distribution (Fig. 1) as posterior, anterior, diffuse, brainstem-cerebellum, or basal ganglia [12]. Lesion severity was categorized as mild, moderate, or severe according to the vasogenic edema extent, the involvement of deep structures (cerebellum, brainstem, or basal ganglia), and the presence of parenchymal hemorrhage [13].

In addition to demographics, from each reported case, we evaluated the following clinical information: non-renal and non-cerebral pre-existing conditions, initial presentation, the abovementioned neurologic findings, blood pressure values, laboratory data (including creatinine among others), patient management, and the disease course. The following neuroimaging data were weighted: brain scans performed with magnetic resonance imaging (MRI) rather than computed tomography (CT), accuracy of technical report, description of distribution patterns, figure and caption preciseness.

Two authors graded subjectively in consensus the comprehensiveness in reporting the clinical (CO, MGB) and the neuroimaging data (RW, MCL) as satisfactory or good.

### Analysis

A pilot-tested form was used for data extraction and recording. Results are given as median and interquartile range or as frequency, as appropriate. The Wilcoxon rank-sum test was used to compare continuous variables and the Fisher exact test to compare dichotomous variables. Significance was assigned at *P* < 0.05.

## Results

### Search outputs

**Fig. 2:**
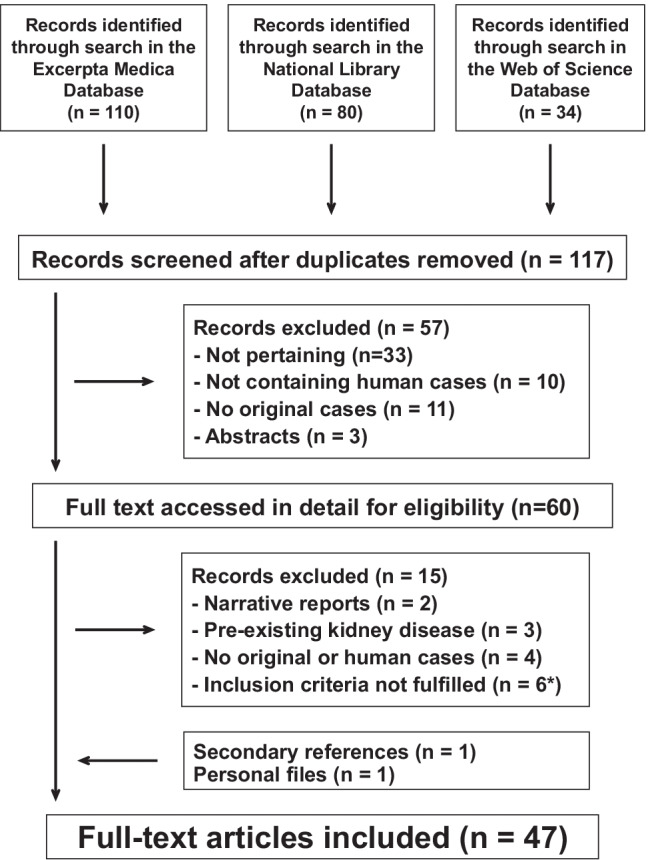
Posterior reversible leukoencephalopathy syndrome associated with acute postinfectious glomerulonephritis. Flowchart of the literature search process (*poor-quality neuroimaging studies, *n* = 5; poorly documented kidney disease, *n* = 1)

The study flowchart is shown in Fig. 2. For the final analysis, we retained 47 scientific reports [4, 5, 14–58] detailing 52 original cases published since 1993: 22 from Asia (Turkey, *n* = 8; India, *n* = 7; Japan, *n* = 3; Pakistan, *n* = 1; Saudi Arabia, *n* = 1; Sri Lanka, *n* = 1, Taiwan, *n* = 1), nine from Europe (Germany, *n* = 2; Italy, *n* = 2; Belgium, *n* = 1; Greece, *n* = 1; Netherlands, *n* = 1; Spain, *n* = 1; Switzerland, *n* = 1), eight from North America (USA, *n* = 7; Canada, *n* = 1), six from South America (Argentina, *n* = 2; Brazil, *n* = 1; Colombia, *n* = 1; Trinidad, *n* = 1; Uruguay, *n* = 1), and two from Africa (Morocco, *n* = 1; Tunisia, *n* = 1). Thirty-eight articles were published in English, five in Spanish, two in French, one in Dutch, and one in German.

The comprehensiveness in reporting clinical data was good in 44 and satisfactory in eight cases, that is, reporting neuroimaging data was good in 31 and satisfactory in 21 cases.

### Clinical and laboratory data at presentation

Information on the 52 patients – 50 children and 2 adults – appears in Table 1. The initial clinical presentation was neurologic in three quarters of the cases. Seizures, headache, nausea, altered mental status, and visual disturbances were, in decreasing order of frequency, the main neurologic features. Arterial hypertension was severe in approximately 80% of cases. Stage 3 acute kidney injury was noted in less than 10% of cases.

**Table 1:** Clinical, laboratory, and imaging data in 52 patients from 7 to 32 years of age with acute poststreptococcal glomerulonephritis associated with posterior reversible leukoencephalopathy syndrome. Data are given as median (with interquartile range) or as relative frequency (with percentage)

	All	Posterior	Diffuse	Brainstem cerebellum	P-value*
***N***	52	28	23	1	
**Males:females, *N***	32:20	18:10	14:9	0:1	> 0.999
**Age**					
Years	11 [9–13]	10 [9–12]	12 [10–14]	9	0.121
≤ 18 years, N (%)	50 (96)	26 (93)	23 (100)	1	0.494
**Pre-existing condition, *N* (%)**	2* (4)	0 (0)	2* (9)	0	0.492
**Initial presentation**					
Neurologic, *N* (%)	37 (71)	21 (75)	16 (70)	0	0.757
Renal, *N* (%)	13 (25)	5 (18)	7 (30)	1	0.336
Neurologic and renal, *N* (%)	2 (4)	2 (7)	0 (0)	0	0.495
**Neurologic features**					
Headache-nausea, *N* (%)	44 (85)	25 (89)	19 (83)	1	0.687
Altered mental status, *N* (%)	38 (73)	20 (71)	18 (78)	0	0.749
Seizures, *N* (%)	46 (88)	23 (82)	22 (96)	1	0.739
Visual abnormalities, *N* (%)	29 (56)	17 (61)	11 (48)	1	0.407
Further focal signs, *N* (%)	13 (25)	5 (18)	8 (35)	0	0.207
**Blood pressure**					
Normal-elevated, *N* (%)	3 (6)	0 (0)	3 (13)	0	0.085
Stage 1 hypertension, *N* (%)	3 (6)	0 (0)	2 (9)	1	0.198
Stage 2 hypertension, *N* (%)	5 (10)	4 (4)	1 (4)	0	0.362
Severe hypertension, *N* (%)	41 (79)	24 (86)	17 (74)	0	0.316
**Acute kidney injury**					
Stage 1, *N* (%)	32 (62)	19 (68)	13 (57)	0	0.561
Stage 2, *N* (%)	16 (31)	8 (29)	8 (35)	0	0.369
Stage 3, *N* (%)	4 (8)	1 (4)	2 (9)	1	0.583
**Neuroimaging severity score**					
Mild, *N* (%)	49 (94)	28 (100)	21 (91)	0	0.198
Moderate, *N* (%)	2 (4)	0 (0)	1 (4)	1	0.451
Severe, *N* (%)	1 (2)	0 (0)	1 (4)	0	0.451
**Drug therapy**					
Antihypertensives, *N* (%)	48 (92)	27 (96)	21 (91)	0	0.583
Antiepileptics, *N* (%)	38 (73)	19 (68)	19 (83)	0	0.336
Corticosteroids, *N* (%)	11 (21)	3 (21)	7 (13)	1	0.154

^*^Posterior versus diffuse

The following neuroimaging techniques were used for the evaluation of disease onset in the 52 patients: MRI (*n* = 24); CT followed by MRI (*n* = 22); CT (*n* = 6). A CT with a contrast agent was obtained in four cases. An invasive digital subtraction angiography study was performed in two cases [17, 27]. The following magnetic resonance imaging sequences were used in the 46 patients: T2WI and/or FLAIR, *n* = 46; DWI with ADC, *n* = 20; TOF, *n* = 3; T1 C + , *n* = 7. A posterior pattern of distribution was detected in 28 cases, a diffuse pattern in 23 cases, and a brainstem-cerebellum-predominant pattern in the remaining case [14]. No patient was found to have an anterior or a basal ganglia pattern. The neuroimaging severity score was mild in about 95% of cases.

Further neuroimaging abnormalities were observed in three cases [32, 33, 56], as shown in Table 2. Two of the mentioned children were concurrently affected by sickle cell anemia [33, 56]. The 9-year-old girl reported by Hanafy [56] concomitantly presented with an acute postinfectious glomerulonephritis and a vaso-occlusive crisis. No features consistent with a vaso-occlusive crisis were noted in the 10-year-old boy reported by Pashankar [33].

**Table 2:** Neuroimaging abnormalities other than vasogenic edema in 3 patients with PRES associated with acute poststreptococcal glomerulonephritis [32, 33, 56]

Gender	Female [56]	Male [33]	Female [32]
Age, years	9	10	13
Concomitant condition	Sickle cell disease with vaso-occlusive crisis	Sickle cell disease without vaso-occlusive crisis	None
Neuroimaging PRES pattern	Diffuse	Diffuse	Diffuse
Blood pressure	Stage 3 hypertension	Stage 3 hypertension	Normal-elevated
Abnormality	Ischemic lesions involving the cortex of the right occipito-parietal lobe and the right thalamic region	Right frontal intraparenchymal hemorrhage	Supratentorial subarachnoid hemorrhage

No significant statistical difference was noted between patients with a posterior pattern and patients with a diffuse pattern with respect to demographics, initial clinical presentation, neurologic features (including visual disturbances), blood pressure, kidney injury, and neuroimaging severity score (Table 1).

Patients with severe arterial hypertension and patients with normal or non-severe hypertension did not significantly differ with respect to gender, age, neurologic features, severity of acute kidney injury, and edema distribution pattern (Table 3). Antihypertensive drugs were more frequently prescribed in the group of patients with severe hypertension.

**Table 3:** Characteristics of patients with severe arterial hypertension and patients with normal or non-severe hypertension. Data are presented as median (with interquartile range) or as frequency (with percentage)

	Blood pressure		*P*-value
	Severe arterial hypertension	Normal or non-severe hypertension	
***N***	41	11	
**Males:females, *N***	25:16	7:4	> 0.999
**Age, years**	11 [9–13]	12 [10–14]	0.208
**Neurologic features**			
Headache-nausea, *N* (%)	35 (85)	9 (82)	> 0.999
Altered mental status, *N* (%)	29 (71)	9 (82)	0.469
Seizures, *N* (%)	37 (90)	9 (82)	0.595
Visual abnormalities, *N* (%)	22 (54)	7 (64)	0.735
Further focal signs, *N* (%)	12 (29)	1 (9.1)	0.253
**Acute kidney injury**			
Stage 1, *N* (%)	25 (61)	7 (64)	> 0.999
Stage 2, *N* (%)	14 (34)	2 (18)	0.468
Stage 3, *N* (%)	2 (4.9)	2 (18)	0.193
**Edema distribution pattern**			
Posterior, *N* (%)	24 (58)	4 (36)	0.308
Diffuse, *N* (%)	17 (41)	6 (55)	0.507
Brainstem-cerebellum, *N* (%)	0	1 (9.1)	0.212
**Drug therapy**			
Antihypertensives, *N* (%)	41 (100)	7 (64)	< 0.005
Antiepileptics, *N* (%)	30 (73)	8 (73)	> 0.999
Corticosteroids, *N* (%)	9 (22)	2 (18)	> 0.999

### Management course

Drug management is depicted in Table 1. Noticing that antihypertensive and antiepileptic drug therapy was administered in a heterogeneous manner, was frequently adjusted, and was often poorly documented in the articles, no attempt was made to condense these data. Eleven patients received systemic corticosteroids.

A full resolution of clinical findings including among others the neurologic examination, blood pressure, urinalysis, and kidney function was documented within 3 to 16 weeks in 47 cases, including the three children found to have either an ischemic or a hemorrhagic brain lesion [32, 33, 56].

Follow-up neuroimaging studies were performed in 38 cases [2–8] 4 weeks after disease onset: MRI in 33 cases (including one patient revalued also by a digital subtraction angiography) and CT in 5 cases. A complete resolution of the lesions was noted in all of them, including the aforementioned three cases complicated by ischemic or hemorrhagic lesions [32, 33, 56].

## Discussion

This is the first systematic review to investigate the association of acute postinfectious glomerulonephritis with PRES. The results indicate that this association predominantly affects, like glomerulonephritis without encephalopathy [6, 7], male school-aged children, that it is associated with severe arterial hypertension in four out of five cases and its prognosis is generally favorable.

It has been suggested that severe acute-onset arterial hypertension accounts for most cases of PRES [1, 2]. In our survey, blood pressure was severely elevated in approximately 80% of cases, thereby supporting the abovementioned link. The term hypertensive encephalopathy was traditionally used to denote this important form of PRES [2, 59, 60]. Since cerebral blood flow is normally maintained at a constant level by vascular resistance autoregulation, PRES likely occurs in cases with blood pressure beyond the upper autoregulation limit. In this setting, a breakdown of the blood–brain barrier allows for the extravasation of blood products and extracellular vasogenic edema, which mainly affects the white matter via leakage of fluid from capillaries [59, 60]. Blood pressure was normal or not severely elevated in approximately 20% of cases included in this analysis [59, 60]. The present figures differ from those observed in immunoglobulin A vasculitis [61]. In the latter condition, PRES was associated with severe hypertension in no more than every third case, suggesting a crucial pathogenic role for the cerebral vasculitis in the majority of cases [61]. It is therefore tempting to assume that, like in immunoglobulin A vasculitis associated with PRES [61], an infectious precursor may concurrently trigger both a glomerulonephritis and a cerebral vascular dysfunction [14]. Agents such as calcineurin inhibitors; sirolimus; platinum-containing cancer drugs; angiogenesis inhibitors; and, more rarely, systemic corticosteroids have also been associated with PRES [3] but none of the patients included in this review were pretreated with the mentioned agents. Very recent data argue for a central role of innate in the pathophysiology of PRES [62].

The term PRES was adopted 25 years ago [1] because neuroimaging studies detected a brain edema situated in the subcortical white matter of the occipital region. This edema pattern was noted in slightly more than half of our cases. In this study, however, we observed a diffuse edema in more than 40% of cases, as reported in the recent literature [2, 12, 13]. It has been therefore suggested to abandon the word “posterior” and use instead terms such as reversible leukoencephalopathy syndrome or acute brain capillary leak syndrome [2]. Interestingly, demographics, the clinical features, and the severity of arterial hypertension and kidney disease did not differ between patients with posterior and diffuse brain edema. Intracranial hemorrhage and ischemia may occur in a minority of patients with PRES, as confirmed by the present analysis [2]. Remarkably, two of the three patients with the aforementioned complications of PRES were affected by sickle cell anemia. Since PRES is being increasingly recognized in sickle cell anemia [63], we tentatively speculate that both acute glomerulonephritis and sickle cell anemia might have contributed to the development of PRES.

The present data do not allow us to suggest the management of PRES in acute postinfectious glomerulonephritis. However, it is assumed but not proven that early recognition and treatment are of paramount importance because PRES is usually reversible with appropriate management [2, 59, 60, 64]. Neuroimaging is essential to the diagnosis. While a brain CT scan is sometimes the first study performed, a brain magnetic resonance imaging with an imaging protocol tailored to PRES is the critical imaging method [65].

The currently recommended mainstay of therapy is reduction of excessive blood pressure. With blood pressure lowered, most patients improve dramatically [64]. Goal blood pressure depends on presenting levels. Like in all cases of severe symptomatic acute hypertension, a reduction of mean blood pressure by ≤ 20–25% within 6–9 h is often advised [63, 64, 66]. However, excessive blood pressure lowering might reduce pressure below the autoregulatory range [64, 66]. Reducing blood pressure is also recommended for lower levels of arterial hypertension. In these cases, a reduction by 10% within days seems a realistic target [64]. The use of easily titratable parenteral agents is effective and safe in reducing blood pressure to a desirable range. Systemic corticosteroids are sometimes prescribed in acute postinfectious glomerulonephritis and in PRES [2, 6, 7]. However, this is not generally recommended.

There are limitations and strengths that should be considered when reading the results of this review. The major limitation results from the small number of reported cases, which were sometimes not very well documented. Second, since treatment recommendations can be uneasily inferred by pooling individual cases, suggested therapy recommendations arise from low-quality evidence. Finally, a systematic review addressing all PRES cases associated with an acute glomerulonephritis syndrome including among others immunoglobulin A vasculitis, immunoglobulin A glomerulonephritis, and classic post-diarrheal hemolytic-uremic syndrome might have perhaps allowed for a more in-depth data analysis.

The most relevant strength of the study relates to the comprehensive and exhaustive literature search, which aimed at surveying the entire literature on PRES complicating acute postinfectious glomerulonephritis.

## Conclusion

Unlike immunoglobulin A vasculitis, severe hypertension is the main factor underlying PRES in acute poststreptococcal postinfectious glomerulonephritis. The mechanisms underlying PRES in the absence of severe hypertension deserve future investigations.

## Supplementary Information

ESM 1 (PPTX 316 kb)
