# Supplementary material for: Posterior reversible leukoencephalopathy syndrome associated with acute postinfectious glomerulonephritis: systematic review
Source: Pediatr Nephrol. 2021 Sep 21;37(4):833–41. doi: 10.1007/s00467-021-05244-z (PMC8960599; doi:10.1007/s00467-021-05244-z)
Supplement: Supplementary file 1 — (PPTX 316 kb) [file 467_2021_5244_MOESM1_ESM.pptx]

## Slide 1
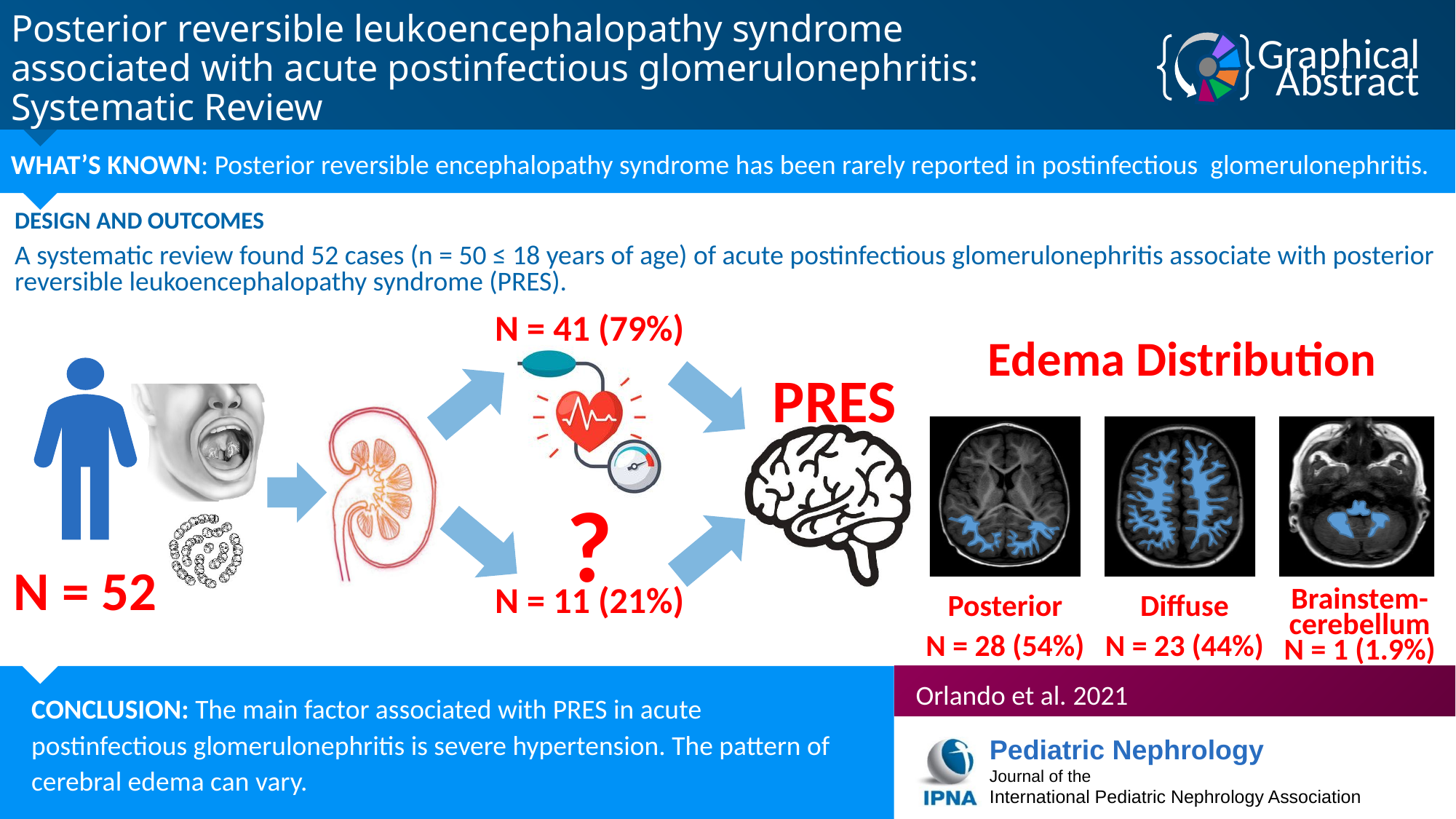

Posterior reversible leukoencephalopathy syndrome associated with acute postinfectious glomerulonephritis: Systematic Review
WHAT’S KNOWN: Posterior reversible encephalopathy syndrome has been rarely reported in postinfectious glomerulonephritis.
DESIGN AND OUTCOMES
A systematic review found 52 cases (n = 50 ≤ 18 years of age) of acute postinfectious glomerulonephritis associate with posterior reversible leukoencephalopathy syndrome (PRES).
N = 41 (79%)
Edema Distribution
PRES
?
N = 52
N = 11 (21%)
Posterior
N = 28 (54%)
Diffuse
N = 23 (44%)
Brainstem-
cerebellum
N = 1 (1.9%)
Orlando et al. 2021
CONCLUSION: The main factor associated with PRES in acute postinfectious glomerulonephritis is severe hypertension. The pattern of cerebral edema can vary.
